# RPT-Mamba: A Range-Aware Physical Token Mamba Network for Far-Field mmWave Radar Gesture Recognition

**DOI:** 10.3390/s26134122

**Published:** 2026-06-30

**Authors:** Yitong Shi, Pei Peng, Zhiyuan Wang

**Affiliations:** 1 School of Communication and Information Engineering, Nanjing University of Posts and Telecommunications, Nanjing 210023, China; 2 School of Electrical and Automation Engineering, Nanjing Normal University, Nanjing 210023, China

**Keywords:** mmWave radar, gesture recognition, sparse point cloud, range generalization, Mamba, attribute reconstruction

## Abstract

Millimeter-wave (mmWave) radar provides a privacy-preserving and illumination-robust sensing modality for contactless gesture recognition. However, sparse radar point clouds degrade substantially as sensing distance increases: the number of valid detections decreases, echo intensity attenuates, and Doppler-related motion cues become less reliable. Such range-induced degradation leads to a distribution shift between near-range training samples and far-field test samples, making it difficult for models trained at short distances to generalize to unseen longer distances. Existing point-cloud gesture recognition methods usually treat radar detections as generic sparse point sequences and rarely model distance-related point loss, echo attenuation, and physical-attribute unreliability explicitly. This work introduces RPT-Mamba, a range-aware physical token Mamba network for sparse mmWave radar point cloud sequences. RPT-Mamba constructs physical point tokens from spatial coordinates, Doppler velocity, echo intensity, point-level range, and sample-level range information. During training, a range-aware stochastic degradation strategy adaptively removes points and masks dynamic attributes according to the estimated sensing distance, while a context-guided attribute reconstruction objective recovers masked Doppler and intensity attributes from spatial and frame-level context. A bidirectional Mamba temporal encoder then models long-range gesture dynamics over frame tokens. On the public mTransSee dataset, RPT-Mamba achieves 92.09% accuracy and 92.04% Macro-F1 under the random split protocol, and 85.34% accuracy and 84.77% Macro-F1 under a challenging near-to-far protocol, exceeding point-cloud, radar-gesture, Transformer, and Mamba baselines.

## 1. Introduction

Gesture recognition is an important natural human–computer interaction technique and has been widely studied for smart homes, in-vehicle interaction, wearable devices, augmented reality, and contactless control. Existing methods mainly rely on RGB cameras, depth cameras, inertial sensors, or wireless sensing devices. Vision-based methods can capture rich appearance and spatial information, but their performance is affected by illumination changes, occlusion, privacy concerns, and complex backgrounds. Wearable-sensor methods often achieve stable recognition accuracy, but require users to wear additional devices, which limits their convenience in natural interaction scenarios. In contrast, mmWave radar is contactless, robust to lighting conditions, privacy preserving, and sensitive to subtle motion; it has therefore become an increasingly important sensing modality for gesture recognition.

An mmWave radar can estimate target range, radial velocity, angle, and echo intensity by transmitting and receiving high-frequency electromagnetic waves. Existing mmWave gesture recognition methods usually use one of two representations. One line of work converts radar returns into image-like features, such as range-Doppler maps, range-time maps, Doppler-time maps, angle-time maps, range-angle maps, or micro-Doppler spectrograms, and then applies conventional machine learning, convolutional neural networks (CNNs), recurrent networks, or Transformer-based backbones. Recent studies show that multifeature radar-map fusion improves robustness in complex scenes [1], and range-Doppler-map-based residual recurrent attention networks have also been explored for gesture recognition [2]. These dense representations are convenient for image backbones, and signal attenuation or weakened responses can still be reflected in radar maps. However, the gridding process aggregates individual detections into discretized cells, making point-level detection loss and per-point attribute reliability less explicit than in point-cloud representations.

The other line of work directly models sparse radar point clouds, where each detected point carries spatial coordinates and physical attributes such as Doppler velocity and echo intensity. Recent mmWave gesture recognition studies have used multiscale point-cloud feature extraction and sparse point-cloud spatio-temporal modeling [3,4]. This paper follows the point-cloud route not because it is simpler, but because it preserves the detection-level structure needed to analyze far-field degradation. In practical gesture recognition, the sensing distance between the user and the radar is often variable. As the distance increases, radar echoes become weaker, valid detections may disappear, and Doppler/intensity attributes become less reliable. These effects manifest in point-cloud representations as point loss, intensity attenuation, and degraded dynamic attributes.

The difficulty of modeling mmWave radar point clouds arises at both the signal and representation levels. At the signal level, recent 4D mmWave radar point-cloud studies report inherent sparsity, noise, limited spatial resolution, weak geometric detail, multipath artifacts, and false targets [5,6]. At the representation level, a gesture sample is an unordered and frame-dependent point sequence: the number of detections changes from frame to frame, while the discriminative motion pattern is distributed over time. Far-field sensing further intensifies this problem by reducing point density and weakening physical attributes. Therefore, robust far-field gesture recognition requires joint modeling of sparse point cloud structure, distance-related physical degradation, and long-range temporal dynamics.

**Figure 1:**
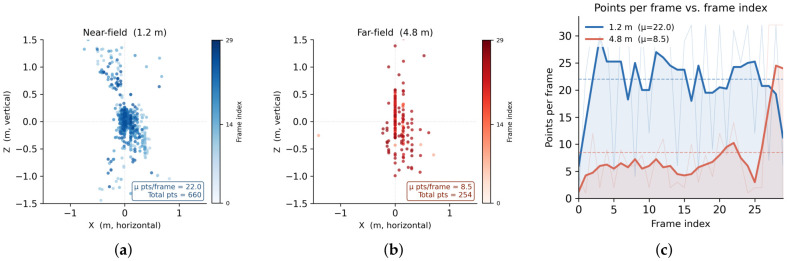
Comparison of the same PL gesture under near-field (1.2 m) and far-field (4.8 m) mmWave radar sensing. Each sample is preprocessed identically to the model input: every 10th raw frame is selected and at most 30 frames are retained (frame indices 0–29 on the color bar). The two point-cloud views share the same coordinate range. At 4.8 m, valid detections become much sparser and the gesture trajectory is less separable. (**a**) Near field (1.2 m). (**b**) Far field (4.8 m). (**c**) Valid points per frame.

Figure 1 illustrates the same gesture under near-field and far-field conditions. The near-field point cloud is dense and structurally clear, whereas the far-field point cloud is sparse and scattered. As shown in the example gesture in Figure 1, the number of valid points per frame decreases from 22.0 at 1.2 m to 8.5 at 4.8 m, approximately 39% of the near-field value. This pronounced density reduction suggests that sensing distance should be treated as an explicit nuisance factor rather than being left to implicit feature learning.

Existing mmWave radar gesture recognition methods can be broadly divided into image-like radar feature modeling and sparse point cloud modeling. Image-like methods make radar processing compatible with mature visual backbones, but gridding can obscure which detections are missing or physically unreliable. Point-cloud methods operate directly on radar detections using PointNet [7], PointNet++ [8], recurrent networks [9], Transformers [10], or state-space models. For example, Pantomime uses PointNet++ and long short-term memory (LSTM) for sparse mmWave point cloud sequences [11]; ST-PCT introduces a point cloud Transformer for mmWave activity sensing [12]; and STPM adopts Mamba for mmWave radar point cloud activity recognition [13]. These methods mainly report in-distribution recognition accuracy, while the distance-induced shifts in point density, echo strength, and attribute reliability are less explicitly modeled.

RPT-Mamba is specifically designed to address this distance-induced distribution shift. Compared with radar-map-based range-robust methods, RPT-Mamba keeps the point-level physical attributes and explicitly models which detections and attributes become unreliable as range increases. Compared with radar point cloud Mamba models such as STPM, which mainly use Mamba as a spatio-temporal sequence encoder, RPT-Mamba differs in three aspects: it constructs physical tokens from spatial coordinates, Doppler velocity, echo intensity, point-level range, and sample-level range; it applies distance-adaptive point dropping and attribute masking as a training-time degradation process; and it uses context-guided reconstruction to regularize the bidirectional Mamba encoder under incomplete far-field observations. A bidirectional Mamba temporal encoder then processes the frame-token sequence, enabling the final classifier to incorporate both the physical structure of individual radar detections and the temporal evolution of the gesture.

The main contributions are as follows:

**C1.**  **Physical point tokenization.** We encode spatial coordinates, Doppler velocity, echo intensity, point-level range, and sample-level range condition, allowing distance information to enter the model before temporal aggregation.
**C2.**  **Range-aware degradation learning.** We link the perturbation strength to the estimated sensing distance. Together with Doppler/intensity reconstruction, this design converts far-field signal attenuation into an explicit training signal rather than a hidden source of distribution shift.
**C3.**  **Near-to-far evaluation protocol.** We construct a near-to-far protocol on mTransSee. Under this setting, RPT-Mamba improves accuracy over STPM from 81.67% to 85.34% and raises the extreme far-field Bucket 3 accuracy from 73.24% to 78.56%.

## 2. Related Work

### 2.1. mmWave Radar Gesture Recognition

Gesture recognition has been extensively studied for natural human–computer interaction. Compared with RGB or depth cameras and wearable inertial sensors, mmWave radar offers contactless sensing, lighting robustness, and better privacy protection. Recent advances in high-frequency sensing and near-field array imaging further show the continuing development of radar-oriented signal processing techniques [14]. Early mmWave radar gesture recognition methods typically transform radar returns into range-Doppler maps, range-angle maps, micro-Doppler spectrograms, or other image-like features, followed by conventional machine learning or convolutional neural networks. Recent radar-map and signal-level methods further combine multiple feature views, cross-modal synthesis, or attention-based temporal modeling to improve robustness [1,2,15]. Such feature maps make radar processing compatible with image backbones, but they also aggregate individual detections, Doppler attributes, and frame-wise point sparsity into dense representations.

With the development of radar point cloud processing, an increasing number of studies have directly used sparse mmWave radar point clouds for gesture recognition. Point cloud sequences preserve spatial position, Doppler velocity, and echo intensity variations during hand motion. The mTransSee dataset and its baseline mSeeNet provide public data and an evaluation basis for environment-independent mmWave gesture recognition [16]. GesturePrint further studies user identification and cross-dataset generalization for mmWave gesture recognition [17]. Pantomime combines PointNet++ and LSTM for sparse radar point cloud gesture recognition [11]. Recent work has also explored traffic-police gestures, 3D point cloud spatiotemporal information, multiscale features, local–global Transformer representations, smart-home sparse point cloud modeling, and lightweight sparse radar point-cloud activity recognition [3,4,18,19,20,21,22,23]. These studies indicate a shift from handcrafted or image-like radar features toward direct point-level modeling.

However, most existing methods focus on conventional in-distribution evaluation and pay insufficient attention to point cloud sparsification, echo attenuation, and distribution shift induced by sensing distance. In practical deployments, the distance between the user and the radar is often variable. Far-field gestures usually produce sparser and noisier point clouds, resulting in measurable performance degradation. Improving model generalization to unseen far-field distances remains a key challenge.

### 2.2. mmWave Point Cloud Sequence Modeling

Point clouds are unordered, sparse, and irregular. For mmWave radar gesture recognition, each sample consists of consecutive frames of sparse detections; a model must extract spatial structure within each frame and dynamic dependencies across frames. In spatial modeling, PointNet first applies deep learning directly to unordered point sets using shared multilayer perceptrons and symmetric aggregation [7]. PointNet++ further introduces local grouping and hierarchical feature extraction [8]. Graph neural networks, point convolutions, and point cloud Transformers have also been used to model local relationships and global dependencies.

In temporal modeling, a common framework first extracts frame-level point cloud features and then uses LSTM [9], temporal CNNs, or Transformers [10] for sequence modeling. LSTM can capture temporal order but is limited in long-range dependency modeling and parallel training. Temporal CNNs are efficient but constrained by kernel sizes. Transformers offer global interaction, but their quadratic complexity can be costly for long sequences. Recent radar activity recognition studies have explored lightweight Transformers, residual attention structures, and point cloud video models to improve dynamic representation [2,24,25].

Point cloud video models and state-space models have further advanced dynamic point cloud recognition. P4Transformer treats point cloud sequences as 4D point cloud data and uses a Transformer to model spatial and temporal relationships [26]. Mamba and related state-space models provide linear-time long-sequence modeling and have been applied to vision, video, and point cloud analysis [27,28,29,30,31,32,33,34,35]. Radar-oriented Mamba studies have also appeared in micro-Doppler activity recognition, mmWave point-cloud activity recognition, and 4D mmWave point-cloud enhancement [6,13,36]. Nevertheless, mmWave radar point clouds differ from LiDAR and depth-camera point clouds because they are much sparser and include physical attributes such as Doppler velocity and echo intensity. A generic point sequence encoder can model temporal order, but it does not explicitly account for the range-dependent reliability of point survival, intensity, or Doppler cues. RPT-Mamba introduces this range-conditioned modeling stage before temporal encoding.

The distinction from existing radar point cloud Mamba and range-robust gesture recognition methods is therefore methodological rather than only architectural. STPM adopts Mamba to model spatial-temporal dependencies in mmWave point clouds, but it does not explicitly encode sample-level sensing range, nor does it construct a distance-conditioned degradation and reconstruction objective. Range-robust radar gesture methods based on radar maps or multifeature fusion can improve robustness to environmental or signal variations, but their dense representations make point survival, Doppler reliability, and echo-intensity attenuation less explicit. In contrast, RPT-Mamba introduces a physical point tokenizer that separates spatial and dynamic attributes while injecting point-level and sample-level range cues; applies a range-aware stochastic degradation strategy that increases point dropping and attribute masking with sensing distance; and uses bidirectional Mamba after frame aggregation to exploit temporal context from both directions. This design makes the temporal encoder aware of range-induced point loss through the training objective, rather than treating Mamba as a generic sequence modeling block.

### 2.3. Auxiliary Reconstruction Learning and State-Space Models

Auxiliary reconstruction learning constructs recovery objectives beyond the main task. Masked autoencoding has been effective in vision and point cloud representation learning [37,38,39,40,41], and robustness-oriented learning objectives have also been studied in time-series anomaly detection and physics-informed learning [42,43]. Existing masking strategies usually include random masking, block masking, and tube masking. They primarily focus on geometric or contextual completion, but rarely consider that mmWave radar attributes have different failure modes: a point may disappear entirely, or it may remain spatially observable while Doppler and intensity become unreliable.

In mmWave radar gesture recognition, sensing distance affects point density, echo intensity, and observable motion trajectories. Conventional random masking treats near-field and far-field samples similarly, although their observation quality differs. Range-aware stochastic degradation instead increases the probability of point dropping and attribute masking with the estimated sample range, matching the empirical decline in valid detections and echo intensity.

Long-sequence modeling is also crucial for dynamic gesture recognition. Mamba is a selective state-space model that performs sequence modeling through input-dependent state updates [27]. Compared with Transformers, Mamba has lower long-sequence complexity; compared with recurrent networks, it supports more efficient parallel training and stronger long-range dependency modeling. Recent point Mamba studies indicate that tokenization, serialization, local geometry modeling, and bidirectional scanning can noticeably affect point cloud performance [30,31,32,33,35]. Efficient sequence and dynamical-system modeling has also been explored in neuromorphic computation and nonlinear multiagent systems [44,45,46]. These observations support the use of Mamba after radar-specific point token construction, rather than as a generic replacement for the temporal encoder.

## 3. Method

### 3.1. Overall Architecture

RPT-Mamba takes sparse frequency-modulated continuous-wave (FMCW) mmWave radar point cloud sequences directly as input. Compared with methods that convert radar observations into time-frequency images or heat maps, this design preserves point-level spatial positions, Doppler velocity, and echo intensity. Given a gesture sample, the input is a sequence of *T* sparse point clouds:

(1) $$P=\left\{P^{1},P^{2},\ldots ,P^{T}\right\},\;P^{t}={\left\{p_{i}^{t}\right\}}_{i=1}^{N_{t}},$$

where $P^{t}$ denotes the point set of frame *t*, $N_{t}$ is the number of detected valid points, and $p_{i}^{t}$ is the *i*th point in frame *t*. Since different frames contain different numbers of points, each frame is padded to a maximum number *N*, and a padding mask $M^{t}\in {\left\{0,1\right\}}^{N}$ is used to mark valid points.

**Figure 2:**
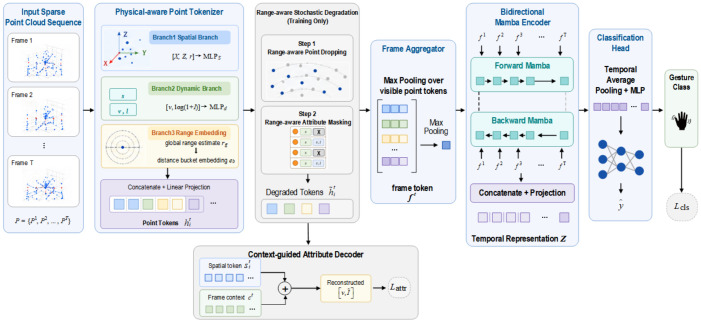
Overall architecture of RPT-Mamba. The framework first constructs physical point tokens, applies range-aware stochastic degradation during training, reconstructs masked Doppler and intensity attributes through a context-guided decoder, aggregates visible point tokens into frame tokens, and finally performs bidirectional temporal modeling with Mamba. Gray modules are used only during training.

At inference time, the main pipeline is expressed as:

(2) $$\hat{y}=\Phi_{\mathrm{cls}}\left(\Phi_{\mathrm{temp}}\left(\Phi_{\mathrm{agg}}\left(\Phi_{\mathrm{tok}}\left(P\right)\right)\right)\right),$$

where $\Phi_{\mathrm{tok}}$ is the physical point tokenizer, $\Phi_{\mathrm{agg}}$ is the frame aggregator, $\Phi_{\mathrm{temp}}$ is the bidirectional Mamba temporal encoder, and $\Phi_{\mathrm{cls}}$ is the classification head. During training, range-aware stochastic degradation and the attribute reconstruction decoder are used to construct an auxiliary loss. These components are removed at inference time and therefore do not increase inference cost. The overall architecture is shown in Figure 2.

### 3.2. Physical-Aware Point Tokenizer

Each mmWave radar detection contains spatial position as well as physical measurements such as Doppler velocity and echo intensity. Under far-field conditions, these components degrade differently: detected spatial locations still provide geometric constraints, while Doppler and intensity are more sensitive to weak echoes and low signal-to-noise ratios. We therefore explicitly encode spatial geometry and dynamic physical attributes through two multilayer perceptron (MLP) branches.

**Range estimation.** To avoid relying on manually annotated sensing distance at inference time, the sample-level range condition is estimated from the input point cloud itself. Because valid points in this dataset mainly lie on the *X*-*Z* plane and the *Y* coordinate varies less due to sensor placement and angular resolution, we approximate point-level range as:

(3) $$r_{i}^{t}=\sqrt{{\left(X_{i}^{t}\right)}^{2}+{\left(Z_{i}^{t}\right)}^{2}}.$$

The median distance over all valid points in a sample is used as the sample-level range estimate:

(4) $$\bar{r}=\mathrm{Median}\left\{r_{i}^{t}|M_{i}^{t}=1,\;1\leq t\leq T\right\}.$$

It is normalized as:

(5) $${\hat{r}}_{g}=\mathrm{clip}\left(\frac{\bar{r}-d_{\min}}{d_{\max}-d_{\min}},0,1\right),$$

where $d_{\min}=1.2\;m$ and $d_{\max}=4.8\;m$ are the minimum and maximum sensing distances covered by the dataset. The operator clip(x,0,1)=min(max(x,0),1) bounds the normalized range to [0,1]. Thus, samples at the nearest supported distance are assigned a degradation severity close to 0, samples at the farthest supported distance are assigned a severity close to 1, and occasional estimates outside the calibrated range are saturated rather than extrapolated. We use the median over valid point-level ranges instead of the mean because sparse radar frames may contain isolated noisy detections or multipath outliers; the median provides a more stable sample-level range estimate for controlling the degradation strength. According to $\bar{r}$, each sample is assigned to one of four distance buckets b∈{0,1,2,3}, corresponding to 1.2–1.8 m, 2.1–2.7 m, 3.0–3.9 m, and 4.2–4.8 m. Each bucket has a learnable range embedding.

**Branch encoding.** For the *i*th valid point in frame *t*, the spatial branch takes $\left[X_{i}^{t},Z_{i}^{t},r_{i}^{t}\right]$ as input:

(6) $$s_{i}^{t}={\mathrm{MLP}}_{s}\left(\left[X_{i}^{t},Z_{i}^{t},r_{i}^{t}\right]\right)\in R^{C/2}.$$

The dynamic branch takes Doppler velocity and log-transformed echo intensity:

(7) $$u_{i}^{t}={\mathrm{MLP}}_{u}\left(\left[v_{i}^{t},{\tilde{I}}_{i}^{t}\right]\right)\in R^{C/2},\;{\tilde{I}}_{i}^{t}=\log\left(1+I_{i}^{t}\right).$$

The logarithmic transform compresses the intensity dynamic range and alleviates the scale difference between strong near-field echoes and weak far-field echoes.

**Figure 3:**
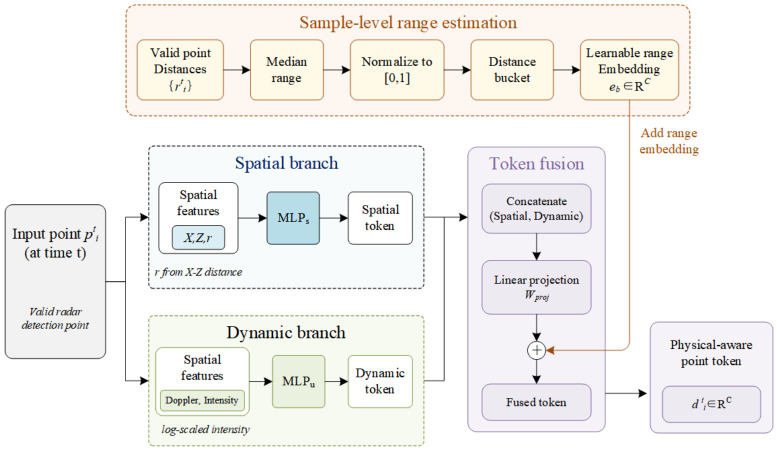
Structure of the Physical-aware Point Tokenizer. Spatial coordinates [X,Z,r] and dynamic attributes $\left[v_{D},\log\left(1+I\right)\right]$ are encoded by two separate multilayer perceptron (MLP) branches. The two branch features are concatenated, linearly projected, and combined with a range-bucket embedding before temporal aggregation.

**Token fusion.** The two branch outputs are concatenated, projected into a *C*-dimensional token space, and augmented with the range-bucket embedding:

(8) $$h_{i}^{t}=W_{\mathrm{proj}}\left[s_{i}^{t}∥u_{i}^{t}\right]+e_{b}\in R^{C},$$

where ∥ denotes feature concatenation and $e_{b}$ is the learnable embedding of bucket *b*. This design allows a point token to jointly encode local spatial structure, dynamic radar attributes, and the sample-level range condition. Figure 3 summarizes the structure of the physical-aware point tokenizer.

### 3.3. Range-Aware Stochastic Degradation

**Figure 4:**
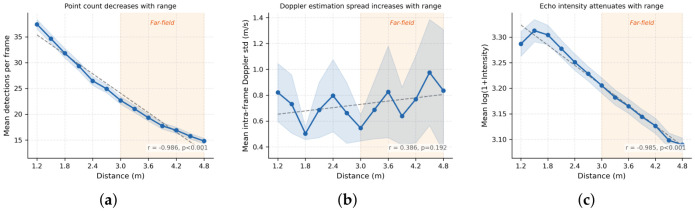
Statistical analysis of mmWave radar point cloud degradation under different sensing distances. The solid blue lines show distance-wise mean values, the blue shaded bands indicate variability around the mean, the gray dashed lines show fitted trends, and the orange shaded regions mark far-field distances. The average number of valid points and average log echo intensity decrease as distance increases, motivating the proposed range-aware stochastic degradation. (**a**) Valid points. (**b**) Doppler std. (**c**) Log intensity.

Far-field radar point clouds primarily exhibit two forms of degradation. First, echo energy attenuates with distance, and weak reflection points may fail to exceed the detection threshold, resulting in fewer valid detections. Second, for retained points, weaker echoes and lower signal-to-noise ratios reduce the reliability of Doppler velocity and echo intensity attributes. Figure 4 reports dataset statistics under different distances. The number of valid points per frame decreases with distance, and the average log intensity also attenuates, confirming substantial far-field degradation.

We formulate range-related degradation as two mutually exclusive training-time perturbations: range-aware point dropping and range-aware attribute masking.

**Range-aware point dropping.** For each valid point, a dropping variable is sampled as:

(9) $$m_{\mathrm{drop},i}^{t}∼\mathrm{Bernoulli}\left(\rho_{\mathrm{drop}}\left({\hat{r}}_{g}\right)\right),$$

where $m_{\mathrm{drop},i}^{t}=1$ means the point is removed from frame aggregation and attribute reconstruction. The dropping probability increases linearly with the normalized range:

(10) $$\rho_{\mathrm{drop}}\left({\hat{r}}_{g}\right)=0.15+0.35{\hat{r}}_{g}.$$

Thus, the dropping probability increases from 0.15 at the nearest range to 0.50 at the farthest range.

**Range-aware attribute masking.** For valid points that are not dropped, an attribute masking variable is sampled as:

(11) $$m_{\mathrm{attr},i}^{t}∼\mathrm{Bernoulli}\left(\rho_{\mathrm{attr}}\left({\hat{r}}_{g}\right)\right),\;\mathrm{if}\;m_{\mathrm{drop},i}^{t}=0,$$

with

(12) $$\rho_{\mathrm{attr}}\left({\hat{r}}_{g}\right)=0.20+0.30{\hat{r}}_{g}.$$

Attribute masking does not remove the detected point. Instead, only the dynamic-branch output is zeroed, while the spatial branch is retained:

(13) $${\tilde{h}}_{i}^{t}=W_{\mathrm{proj}}\left[s_{i}^{t}∥0_{C/2}\right]+e_{b}.$$

This approximates the situation in which Doppler velocity and echo intensity become unreliable under weak far-field echoes.

The perturbed point token during training is therefore:

(14) $${\tilde{h}}_{i}^{t}=\left\{\begin{array}{cc} \mathrm{invalid}, & m_{\mathrm{drop},i}^{t}=1,\\ W_{\mathrm{proj}}\left[s_{i}^{t}∥0_{C/2}\right]+e_{b}, & m_{\mathrm{drop},i}^{t}=0,\;m_{\mathrm{attr},i}^{t}=1,\\ h_{i}^{t}, & m_{\mathrm{drop},i}^{t}=0,\;m_{\mathrm{attr},i}^{t}=0. \end{array}\right.$$

To avoid degenerate frames, at least one original valid point is kept in each non-empty frame during training. If a raw frame has no valid points, a learnable empty-frame token is used.

**Figure 5:**
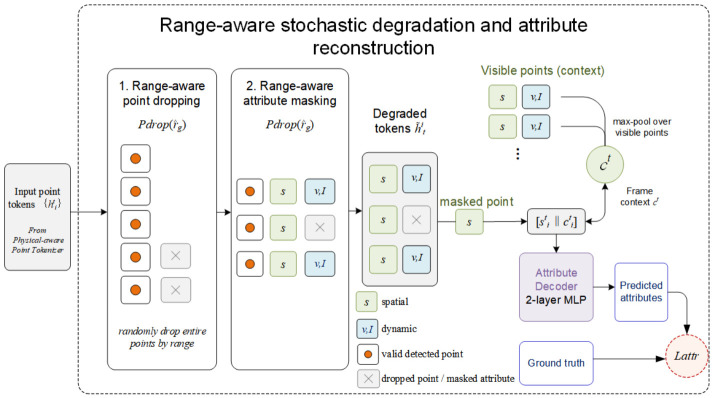
Training-time range-aware stochastic degradation and context-guided attribute reconstruction. As the estimated sample-level range increases, point dropping and attribute masking probabilities increase linearly.

Figure 5 illustrates the training-time degradation and attribute reconstruction process.

### 3.4. Context-Guided Attribute Reconstruction

Range-aware attribute masking creates an auxiliary recovery task for Doppler velocity and echo intensity. The target set of masked attributes is:

(15) $$M_{a}=\left\{\left(t,i\right)|M_{i}^{t}=1,\;m_{\mathrm{drop},i}^{t}=0,\;m_{\mathrm{attr},i}^{t}=1\right\}.$$

For $\left(t,i\right)\in M_{a}$, the spatial branch $s_{i}^{t}$ is retained while the dynamic branch $u_{i}^{t}$ is zeroed. The model must reconstruct the original dynamic attributes from the point location and visible frame context.

The visible points in frame *t* are:

(16) $$\Omega_{t}^{\mathrm{vis}}=\left\{j|M_{j}^{t}=1,\;m_{\mathrm{drop},j}^{t}=0,\;m_{\mathrm{attr},j}^{t}=0\right\}.$$

Their perturbed tokens are max-pooled to obtain the frame-level context:

(17) $$c^{t}=\max_{j\in \Omega_{t}^{\mathrm{vis}}}{\tilde{h}}_{j}^{t}\in R^{C}.$$

For each masked point, the decoder predicts the standardized Doppler and log-intensi-ty attributes:

(18) $${\hat{a}}_{i}^{t}=\Phi_{\mathrm{dec}}\left(\left[s_{i}^{t}∥c^{t}\right]\right)\in R^{2},$$

where $\Phi_{\mathrm{dec}}$ is a two-layer MLP. The mean and standard deviation used for standardizing Doppler velocity and log-intensity are computed only from the training split of each protocol and then applied to the validation and test sets. We let the standardized ground-truth attributes be

(19) $$a_{i}^{t,*}=\left[v_{i}^{t,*},{\tilde{I}}_{i}^{t,*}\right].$$

The reconstruction loss is:

(20) $$L_{\mathrm{attr}}=\frac{1}{|M_{a}|}\sum_{\left(t,i\right)\in M_{a}}{∥{\hat{a}}_{i}^{t}-a_{i}^{t,*}∥}_{2}^{2}.$$

This auxiliary objective encourages the representation to capture the relationship among spatial position, frame-level context, and radar physical attributes. The decoder is used only during training.

### 3.5. Frame Aggregation and Bidirectional Temporal Mamba Encoding

#### 3.5.1. Frame Aggregation

After point token construction and training-time degradation, each frame contains a set of visible point tokens. Given the limited number of valid points in far-field frames and the instability of local point structures at long range, max pooling is adopted as an order-invariant frame aggregation operation. For frame *t*, the retained visible set is:

(21) $$\Omega_{t}=\left\{i|M_{i}^{t}=1,\;m_{\mathrm{drop},i}^{t}=0\right\}.$$

The frame-level representation is:

(22) $$f^{t}=\left\{\begin{array}{cc} \max_{i\in \Omega_{t}}{\tilde{h}}_{i}^{t}, & |\Omega_{t}|\;>0,\\ f_{\mathrm{empty}}, & |\Omega_{t}|\;=0, \end{array}\right.$$

where $f_{\mathrm{empty}}\in R^{C}$ is a learnable empty-frame token. The entire gesture is represented as a frame-token sequence:

(23) $$F=\left[f^{1},f^{2},\ldots ,f^{T}\right]\in R^{T\times C}.$$

#### 3.5.2. Bidirectional Mamba Temporal Encoder

Gestures contain ordered phases such as initiation, primary motion, and completion, and discriminative frames may appear only locally in the sequence. Since the task is offline sequence classification, the full gesture sequence is available at inference time. We therefore adopt a bidirectional Mamba encoder to use both past and future motion context.

We let $F^{\left(0\right)}=F$. For the *l*th bidirectional Mamba layer, the input is first normalized:

(24) $${\bar{F}}^{\left(l-1\right)}=\mathrm{LN}\left(F^{\left(l-1\right)}\right).$$

Forward and backward Mamba encoders are then applied:

(25) $$Z_{f}^{\left(l\right)}={\mathrm{Mamba}}_{f}^{\left(l\right)}\left({\bar{F}}^{\left(l-1\right)}\right)\in R^{T\times C},$$

(26) $$Z_{b}^{\left(l\right)}=\mathrm{Reverse}\left({\mathrm{Mamba}}_{b}^{\left(l\right)}\left(\mathrm{Reverse}\left({\bar{F}}^{\left(l-1\right)}\right)\right)\right)\in R^{T\times C}.$$

The two directions are concatenated and fused:

(27) $$Z^{\left(l\right)}=W_{o}^{\left(l\right)}\left[Z_{f}^{\left(l\right)}∥Z_{b}^{\left(l\right)}\right]\in R^{T\times C},$$

where $W_{o}^{\left(l\right)}\in R^{C\times 2C}$. The layer output is obtained through a residual connection:

(28) $$F^{\left(l\right)}=F^{\left(l-1\right)}+Z^{\left(l\right)},\;l=1,2,\ldots ,L.$$

After *L* layers, the final temporal representation is:

(29) $$Z^{\mathrm{out}}=F^{\left(L\right)}=\left[z_{1}^{\mathrm{out}},z_{2}^{\mathrm{out}},\ldots ,z_{T}^{\mathrm{out}}\right]\in R^{T\times C}.$$

### 3.6. Classification Head and Training Objective

The sequence-level representation is obtained by temporal average pooling:

(30) $$z_{\mathrm{seq}}=\frac{1}{T}\sum_{t=1}^{T}z_{t}^{\mathrm{out}}\in R^{C}.$$

The classification head maps it to gesture logits:

(31) $$\hat{y}=\Phi_{\mathrm{cls}}\left(z_{\mathrm{seq}}\right)\in R^{K},$$

where K=5 in our experiments. The classification loss is cross entropy:

(32) $$L_{\mathrm{cls}}=-\sum_{k=1}^{K}y_{k}\log\left(\mathrm{Softmax}{\left(\hat{y}\right)}_{k}\right).$$

The final training objective is:

(33) $$L=L_{\mathrm{cls}}+\lambda L_{\mathrm{attr}},$$

where λ controls the relative contribution of the auxiliary attribute reconstruction objective. Since the primary task is gesture classification, the reconstruction term is used as a regularizer rather than as an equally weighted objective. We set λ=0.1 for all experiments based on validation-set model selection: this value keeps the Doppler/intensity reconstruction signal active while preventing the auxiliary mean-squared error from dominating the cross-entropy classification loss, especially in early training when reconstructed attributes are still inaccurate. The same value is used across all protocols and ablation experiments. The model is trained end-to-end with AdamW, an initial learning rate of $1\times 10^{-3}$, cosine learning-rate scheduling, and 100 epochs.

## 4. Experiments

### 4.1. Dataset and Evaluation Protocols

We evaluate RPT-Mamba on the public mTransSee mmWave radar gesture dataset [16]. The dataset contains five gesture classes, denoted as CR, KO, PL, PS, and UP. It was collected from 32 subjects under different sensing distances and environmental conditions. After segmenting continuous CSV point cloud sequences based on missing-frame boundaries, we obtain 58,491 gesture samples. The sensing distance covers 13 positions from 1.2 m to 4.8 m at 0.3 m intervals.

Each radar point is represented as:

(34) $$(\mathrm{FrameID},N_{\mathrm{obj}},x,y,z,v,s),$$

where *x*, *y*, and *z* are coordinates, *v* is Doppler radial velocity, and *s* is echo intensity. In the model input, each point is represented as:

(35) $$p_{t,i}=\left[x_{t,i},z_{t,i},v_{t,i},\log\left(1+s_{t,i}\right),r_{t,i},d_{\mathrm{norm}}\right],$$

where $r_{t,i}=\sqrt{x_{t,i}^{2}+z_{t,i}^{2}}$ and

(36) $$d_{\mathrm{norm}}=\mathrm{clip}\left(\frac{\bar{r}-1.2}{4.8-1.2},0,1\right).$$

Each sample is formed by selecting every 10th raw radar frame (stride = 10) and retaining at most T=30 frames; each frame contains at most N=32 points. Frames with more than *N* points are randomly subsampled, while frames with fewer points are zero padded with a padding mask. Each sample is therefore represented as $X\in R^{T\times N\times 6}$.

We use three evaluation protocols. First, the random split protocol evaluates in-distribution performance. Samples are randomly split into training, validation, and test sets with a ratio of 70%, 10%, and 20%, respectively (Table 1).

Second, we introduce a subject-independent evaluation protocol to assess cross-subject generalization and reduce the risk of subject-level data leakage. Unlike sample-wise random splitting, this protocol partitions the dataset at the subject level. All samples from the same subject are assigned exclusively to the training, validation, or test set, ensuring that the subjects in the three subsets are mutually disjoint. Specifically, we perform 5-fold subject-independent cross-validation over the 32 subjects. The subjects are divided into five non-overlapping folds, and each fold is used once as the held-out test subject group. In each round, the remaining subjects are further divided into training and validation subjects without subject overlap. The final performance is reported as the mean and standard deviation of Accuracy and Macro-F1 over the five subject folds.

Third, we design a near-to-far protocol to evaluate far-field generalization. Samples are divided into four distance buckets (Table 2). In this protocol, models are trained and validated only on Bucket 0 and Bucket 1, and tested on the unseen far-field Bucket 2 and Bucket 3 (Table 3).

We report Accuracy and Macro-F1 as the main metrics. Unless otherwise specified, methods compared under the same protocol use identical preprocessing, input settings, and training configurations. For the random split and near-to-far protocols, each experiment is repeated three times, and average results are reported. For the subject-independent protocol, the mean and standard deviation are reported across the five subject folds; the standard deviation reflects fold-to-fold variation rather than repeated-run variation.

### 4.2. Comparison with Existing Methods

#### 4.2.1. Compared Methods

The comparison covers three types of baselines. mSeeNet and GesturePrint are directly related to the mTransSee/mmWave gesture setting [16,17]. Pantomime and STPM represent sparse radar point cloud recognition with PointNet++-LSTM and Mamba-style temporal modeling, respectively [11,13]. PointNet++-LSTM, P4Transformer, and Vanilla Mamba are included to examine whether generic point cloud sequence encoders are sufficient after the input is adapted to mTransSee [8,9,26,27].

#### 4.2.2. Random Split Protocol

Under random splitting, RPT-Mamba reaches 92.09% accuracy and 92.04% Macro-F1 (Table 4). STPM is the closest baseline at 90.34% accuracy, while Vanilla Mamba reaches 88.52%. The gap between RPT-Mamba and Vanilla Mamba suggests that the performance improvement cannot be attributed solely to the use of a Mamba temporal module; distance-aware token construction and training-time degradation also contribute to the final performance.

#### 4.2.3. Subject-Independent Protocol

To further examine whether the model generalizes to unseen subjects, we conduct five-fold subject-independent cross-validation. As shown in Table 5, the evaluation is performed at the subject level, and no subject appears in more than one subset within the same fold. This setting is stricter than sample-wise random splitting because the test samples are collected from subjects that are not observed during training or validation.

Compared with the random split protocol, the subject-independent results show lower recognition accuracy for all methods, indicating that sample-wise splitting may lead to optimistic performance estimates when subject identities are shared across the training and test sets. Nevertheless, RPT-Mamba still achieves the best average performance among the compared methods, with 88.42% Accuracy and 88.01% Macro-F1. The improvement over STPM suggests that the proposed range-aware physical tokenization and degradation-aware training remain effective when the test subjects are unseen during training. In addition, the relatively small standard deviation across the five folds indicates that the proposed method provides stable cross-subject generalization.

#### 4.2.4. Near-to-Far Protocol

Under the near-to-far protocol, all methods show lower accuracy than under random splitting (Table 6). This performance reduction is consistent with the fact that the test samples are drawn from 3.0–4.8 m, a distance range entirely excluded from training. RPT-Mamba reaches 85.34% accuracy and 84.77% Macro-F1, improving over STPM by 3.67 percentage points in accuracy and over Vanilla Mamba by 7.88 percentage points.

#### 4.2.5. Far-Field Distance Bucket Analysis

Table 7 separates the near-to-far test set into Bucket 2 and Bucket 3. Bucket 3 is the more challenging subset because the sensing distance reaches 4.2–4.8 m. In this bucket, RPT-Mamba achieves 78.56% accuracy, compared with 73.24% for STPM and 69.73% for Vanilla Mamba. This performance margin exceeds that observed under the random split protocol, which is consistent with the range-conditioned design being particularly effective when the point cloud is severely degraded.

### 4.3. Computational Complexity and Efficiency Analysis

To further evaluate the deployability of RPT-Mamba, we compare its computational complexity with representative sequence-modeling baselines. All methods are profiled under the same input setting, with an input tensor size of 1×30×32×6. The parameter count and floating-point operations (FLOPs) are computed for a single forward pass under the same implementation and profiling environment, and inference latency is measured on an NVIDIA GeForce RTX 5090 GPU.

As shown in Table 8, RPT-Mamba achieves a favorable balance between recognition performance and computational cost. Compared with STPM, the proposed method reduces the parameter count, FLOPs, and inference latency while providing better near-to-far recognition accuracy. Compared with P4Transformer, RPT-Mamba has fewer parameters and lower latency, although its FLOPs are moderately higher due to the range-aware physical tokenization and bidirectional temporal modeling. Vanilla Mamba has the lowest latency, but it does not explicitly model distance-induced point loss and physical-attribute degradation. Together with the near-to-far recognition results, the complexity comparison suggests that RPT-Mamba remains practical for radar gesture recognition scenarios where both far-field robustness and inference efficiency are required, which is also relevant to reliability-sensitive and resource-constrained hardware systems [47].

### 4.4. Ablation Study and Module Analysis

All ablation experiments are conducted under the near-to-far protocol, which better reveals the effect of each component on far-field generalization.

#### 4.4.1. Key Module Ablation

Table 9 reports key module ablations. The baseline uses the same point encoder and mean pooling without attribute reconstruction, range-aware degradation, or bidirectional Mamba. Attribute reconstruction raises accuracy from 50.37% to 73.84%, indicating that Doppler/intensity recovery provides an effective physical constraint. The third variant applies range-aware point dropping and attribute masking, but removes the reconstruction loss and still uses mean pooling. Its 68.62% accuracy is therefore not directly comparable to random attribute masking under identical controls. Instead, it indicates that range-aware degradation alone does not outperform reconstruction-based learning without temporal modeling. Adding bidirectional Mamba raises accuracy to 85.34%, indicating that the degradation objective requires a temporal encoder capable of exploiting the remaining motion context.

These ablation results indicate that RPT-Mamba is sensitive to drastic architectural simplifications because the proposed components address different aspects of far-field degradation. Attribute reconstruction provides a physical constraint for recovering unreliable Doppler and intensity cues, whereas range-aware degradation exposes the model to distance-dependent point loss and attribute corruption. However, degradation alone may remove useful observations without providing a recovery objective, which explains why the degradation-only variant does not achieve the best performance. Bidirectional Mamba further allows the model to exploit temporal context from the complete gesture sequence, which is particularly important when far-field frames contain sparse or missing detections. Therefore, the performance drop under simplified variants reflects the complementary roles of the proposed modules rather than an isolated dependence on a single component.

#### 4.4.2. Masking and Degradation Strategies

Table 10 compares different masking and degradation strategies under the same full downstream setting: the context-guided reconstruction loss and bidirectional Mamba encoder are both enabled, and only the masking/degradation rule is changed. Random Attribute Masking reaches 79.53% accuracy. Frame-wise and Tube Masking improve this to 81.27% and 82.43%, respectively, by adding temporal structure to the missing pattern. Range-aware Degradation reaches 85.34%, indicating that matching the perturbation pattern to sensing distance is more effective than applying a distance-agnostic mask when the reconstruction and temporal modules are held fixed.

#### 4.4.3. Temporal Modeling Modules

Table 11 isolates the temporal module while keeping the point tokenizer, degradation strategy, and input features unchanged. Mean pooling reaches 68.62% accuracy, confirming that frame-level statistics are insufficient for far-field gestures. LSTM and Transformer improve the result to 73.47% and 79.23%. Vanilla Mamba reaches 82.61%, and bidirectional Mamba further reaches 85.34%, indicating that future context contributes to offline gesture sequences whose discriminative motion may occur near the middle or end of the sample.

#### 4.4.4. Input Feature Ablation

Table 12 studies the contribution of physical input features. Coordinates alone achieve 61.43% accuracy. Adding Doppler raises accuracy to 72.57%, indicating the importance of radial motion. Echo intensity adds another 4.27 percentage points, and point-level range raises the result to 81.96%. The sample-level normalized range further improves the final accuracy to 85.34%, supporting the choice of including both local and global range cues.

### 4.5. Visualization Analysis

#### 4.5.1. Qualitative Degradation and Reconstruction Visualization

**Figure 6:**
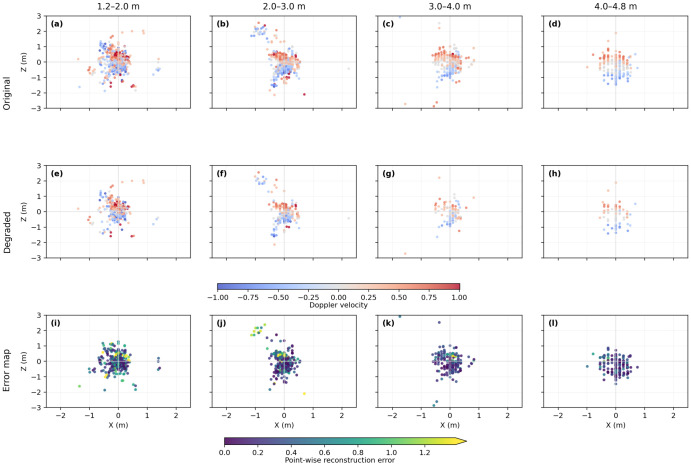
Qualitative visualization of range-aware stochastic degradation and context-guided attribute reconstruction. Columns correspond to four representative distance ranges from near-field to far-field: 1.2–2.0 m, 2.0–3.0 m, 3.0–4.0 m, and 4.0–4.8 m. Panels (**a**–**d**) show the original point clouds, where color indicates Doppler velocity. Panels (**e**–**h**) show the degraded point clouds after applying the proposed range-aware point dropping and attribute masking. Panels (**i**–**l**) visualize the point-wise reconstruction error of the masked dynamic attributes at the attribute-masked point locations. For these points, the spatial coordinates are retained, while the Doppler velocity and echo intensity are masked and predicted from the remaining visible frame context. The visualization illustrates that the proposed degradation strategy produces progressively sparser observations for farther distance ranges, while the reconstruction branch encourages the model to infer physical attributes from incomplete point-cloud context. The error maps are provided for qualitative interpretation rather than distance-wise performance ranking.

Figure 6 provides qualitative visualizations of the proposed range-aware stochastic degradation and context-guided attribute reconstruction.

The original point clouds in panels (a–d) show representative samples from different distance ranges, while panels (e–h) present the corresponding degraded point clouds. As the distance increases, the degraded samples become progressively sparser, which is consistent with the distance-adaptive design of the degradation module. This process simulates the range-dependent sparsity and partial observation common in far-field mmWave radar sensing.

The third row of Figure 6 visualizes the point-wise reconstruction error of the masked dynamic attributes at the attribute-masked point locations. During training, these points retain their spatial coordinates, while their Doppler velocity and echo intensity are masked. The reconstruction head predicts the masked dynamic attributes from the retained spatial information and the visible frame context. Therefore, the reconstruction objective does not aim to recover dropped points or reconstruct complete point clouds during inference; instead, it regularizes the encoder to learn context-aware and range-robust representations from incomplete physical attributes.

#### 4.5.2. Confusion Matrix Analysis

**Figure 7:**
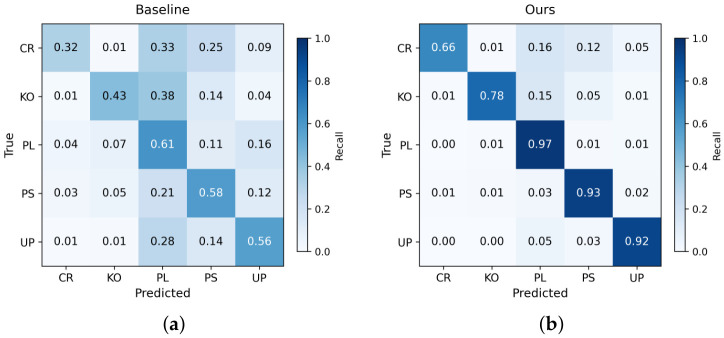
Confusion matrices of the baseline and RPT-Mamba under the near-to-far protocol. Gesture classes are ordered as CR, KO, PL, PS, and UP; rows denote ground-truth labels and columns denote predicted labels. (**a**) Baseline. (**b**) RPT-Mamba.

Figure 7 compares the confusion matrices of the baseline and RPT-Mamba under the near-to-far protocol. The baseline shows substantial inter-class confusion under far-field conditions, especially among gestures with similar motion amplitudes and trajectories. RPT-Mamba produces more concentrated diagonal responses across all five classes, indicating that range-aware degradation, attribute reconstruction, and bidirectional temporal modeling reduce far-field misclassification.

#### 4.5.3. Distance Generalization Visualization

**Figure 8:**
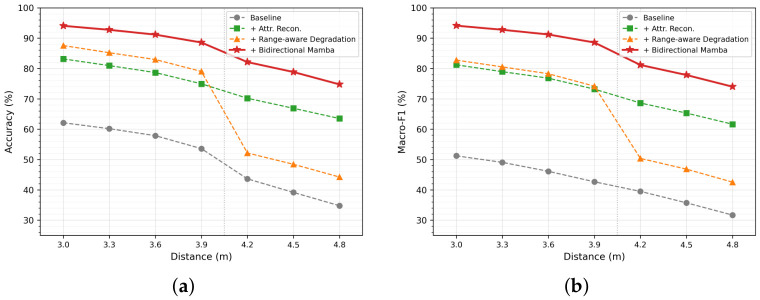
Recognition performance curves of different variants under far-field distances. The horizontal axis is the held-out test distance from 3.0 m to 4.8 m, and the vertical axes show Accuracy and Macro-F1. (**a**) Accuracy vs. distance. (**b**) Macro-F1 vs. distance.

Figure 8 visualizes Accuracy and Macro-F1 over far-field test distances. Table 13 summarizes the bucket-level performance of key variants. All variants degrade from Bucket 2 to Bucket 3, but the degradation patterns differ. Attribute Reconstruction improves Bucket 3 accuracy from 39.18% to 66.85%. The Range-aware Degradation variant corresponds to the same intermediate setting as in Table 9: it introduces distance-conditioned dropping/masking but does not yet include the final bidirectional temporal encoder. It improves Bucket 2 accuracy to 83.67%, whereas Bucket 3 accuracy remains 48.26%. The full model reaches 91.62% on Bucket 2 and 78.56% on Bucket 3, suggesting that distance-aware perturbation and bidirectional temporal modeling should be used jointly to achieve substantial far-field gains.

## 5. Conclusions

RPT-Mamba targets the failure mode in which mmWave radar gesture point clouds become sparse and physically unreliable at longer sensing distances. It encodes each detection with spatial, Doppler, intensity, and range cues; uses range-conditioned point dropping and attribute masking to mimic distance-related degradation; reconstructs masked Doppler and intensity values as an auxiliary constraint; and applies bidirectional Mamba to the resulting frame-token sequence. On mTransSee, RPT-Mamba achieves 92.09% accuracy and 92.04% Macro-F1 under the random split protocol and maintains 88.42% accuracy under subject-independent 5-fold cross-validation. In the near-to-far setting, accuracy increases from 81.67% for STPM to 85.34%, and extreme far-field Bucket 3 accuracy increases from 73.24% to 78.56%. These results indicate that far-field performance benefits from explicit range-conditioned degradation modeling in addition to enhanced temporal encoding. Future work will consider broader deployment scenarios, additional radar configurations, and cross-device generalization.

## Figures and Tables

**Table 1:** Random split protocol on mTransSee. Samples are randomly divided into training, validation, and test sets for in-distribution evaluation.

Set	Samples	Ratio	Usage
Training	40,944	70%	Model parameter learning
Validation	5849	10%	Model selection and hyperparameter tuning
Test	11,698	20%	In-distribution performance evaluation

**Table 2:** Distance bucket definition used in the near-to-far protocol. Buckets 0–1 are used for training/validation, and Buckets 2–3 are held out for far-field testing.

Bucket	Actual Distances	Description
Bucket 0	1.2, 1.5, 1.8 m	Near field
Bucket 1	2.1, 2.4, 2.7 m	Middle field
Bucket 2	3.0, 3.3, 3.6, 3.9 m	Far field
Bucket 3	4.2, 4.5, 4.8 m	Extreme far field

**Table 3:** Near-to-far cross-distance protocol. Models are trained and validated on 1.2–2.7 m samples and tested only on unseen 3.0–4.8 m far-field samples.

Set	Distances	Samples	Usage
Training	1.2–2.7 m	24,225	Model parameter learning
Validation	1.2–2.7 m	2692	Model selection and hyperparameter tuning
Test	3.0–4.8 m	31,574	Far-field generalization evaluation

**Table 4:** Performance comparison under the random split protocol. Accuracy and Macro-F1 are reported on the held-out test set using the same preprocessing and input configuration. Bold values indicate the best performance.

Method	Main Structure	Temporal Modeling	Citation	Accuracy (%)	Macro-F1 (%)
PointNet++-LSTM	PointNet++	LSTM	[8,9]	85.62	85.14
P4Transformer	Point Transformer	Transformer	[26]	89.17	88.63
mSeeNet	Radar sequence model	Temporal modeling	[16]	87.83	87.31
GesturePrint	Radar feature learning	Sequence modeling	[17]	83.45	82.91
Pantomime	PointNet++	LSTM	[11]	84.71	84.23
STPM	Point encoder	Mamba	[13]	90.34	89.87
Vanilla Mamba	Point encoder	Mamba	[27]	88.52	88.04
RPT-Mamba	RPT-Mamba	Bidirectional Mamba	Ours	**92.09**	**92.04**

**Table 5:** Subject-independent 5-fold cross-validation results. The mean and standard deviation are computed across the five subject folds. Bold values indicate the best performance.

Method	Accuracy (%, Mean ± Std)	Macro-F1 (%, Mean ± Std)
P4Transformer	84.38±1.92	83.91±2.05
Vanilla Mamba	85.21±1.74	84.68±1.89
STPM	86.73±1.56	86.18±1.68
RPT-Mamba	88.42±1.35	88.01±1.47

**Table 6:** Performance comparison under the near-to-far protocol. All methods are trained on 1.2–2.7 m samples and evaluated on unseen 3.0–4.8 m samples. Bold values indicate the best performance.

Method	Training Distances	Test Distances	Citation	Accuracy (%)	Macro-F1 (%)
PointNet++-LSTM	1.2–2.7 m	3.0–4.8 m	[8,9]	74.31	73.87
P4Transformer	1.2–2.7 m	3.0–4.8 m	[26]	78.94	78.42
mSeeNet	1.2–2.7 m	3.0–4.8 m	[16]	76.58	76.09
GesturePrint	1.2–2.7 m	3.0–4.8 m	[17]	71.23	70.68
Pantomime	1.2–2.7 m	3.0–4.8 m	[11]	72.85	72.31
STPM	1.2–2.7 m	3.0–4.8 m	[13]	81.67	81.14
Vanilla Mamba	1.2–2.7 m	3.0–4.8 m	[27]	77.46	76.93
RPT-Mamba	1.2–2.7 m	3.0–4.8 m	Ours	**85.34**	**84.77**

**Table 7:** Performance comparison on far-field distance buckets. Bucket 2 covers 3.0–3.9 m and Bucket 3 covers 4.2–4.8 m. Bold values indicate the best performance.

Method	Bucket 2 Acc. (%)	Bucket 2 Macro-F1 (%)	Bucket 3 Acc. (%)	Bucket 3 Macro-F1 (%)
PointNet++-LSTM	80.34	79.83	66.51	66.02
P4Transformer	84.87	84.35	71.16	70.62
mSeeNet	82.61	82.07	68.42	67.89
GesturePrint	77.42	76.89	62.93	62.41
Pantomime	79.08	78.55	64.87	64.34
STPM	87.93	87.41	73.24	72.68
Vanilla Mamba	83.45	82.91	69.73	69.18
RPT-Mamba	**91.62**	**91.65**	**78.56**	**77.66**

**Table 8:** Computational complexity and latency comparison under the same input setting. Parameters, FLOPs, and single-sample inference latency are measured for an input tensor of 1×30×32×6.

Method	Input Size	Params (M)	FLOPs (G)	Latency (ms)
P4Transformer	1×30×32×6	0.803	1.351	0.749
Vanilla Mamba	1×30×32×6	0.404	3.394	0.469
STPM	1×30×32×6	0.806	3.421	1.044
RPT-Mamba	1×30×32×6	0.630	1.791	0.675

**Table 9:** Key module ablation results under the near-to-far protocol. The table isolates the effects of attribute reconstruction, range-aware degradation, and bidirectional Mamba. Bold values indicate the best performance.

Variant	Auxiliary Strategy	Temporal Aggregation	Accuracy (%)	Macro-F1 (%)
Baseline	None	Mean pooling	50.37	42.63
+Attribute Reconstruction	Random masking + reconstruction	Mean pooling	73.84	72.16
+Degradation Only	Range-aware degradation, no reconstruction	Mean pooling	68.62	65.31
+Bidirectional Mamba	Range-aware degradation + reconstruction	Bidirectional Mamba	**85.34**	**84.77**

**Table 10:** Effect of different masking and degradation strategies under the near-to-far protocol. The reconstruction branch and bidirectional Mamba encoder are kept fixed. Bold values indicate the best performance.

Strategy	Accuracy (%)	Macro-F1 (%)
Random Attribute Masking	79.53	78.94
Frame-wise Masking	81.27	80.65
Tube Masking	82.43	81.82
Range-aware Degradation	**85.34**	**84.77**

**Table 11:** Effect of different temporal modeling modules under the near-to-far protocol. The point tokenizer, degradation strategy, and input features are kept unchanged. Bold values indicate the best performance.

Temporal Module	Accuracy (%)	Macro-F1 (%)
Mean Pooling	68.62	65.31
LSTM	73.47	72.81
Transformer	79.23	78.65
Vanilla Mamba	82.61	82.04
Bidirectional Mamba	**85.34**	**84.77**

**Table 12:** Input feature ablation results under the near-to-far protocol. Features are added progressively from spatial coordinates to the full range-aware physical token. Bold values indicate the best performance.

Input Feature Setting	Feature Dim.	Accuracy (%)	Macro-F1 (%)
Coordinates (x,z)	2	61.43	57.82
Coordinates + Doppler	3	72.57	71.24
Coordinates + Doppler + intensity	4	76.84	75.93
Coordinates + Doppler + intensity + point-level range	5	81.96	81.33
Full features (+sample-level normalized range)	6	**85.34**	**84.77**

**Table 13:** Recognition performance of variants on far-field distance buckets. B2 and B3 denote Bucket 2 and Bucket 3, respectively. Bold values indicate the best performance.

Variant	B2 Acc. (%)	B2 F1 (%)	B3 Acc. (%)	B3 F1 (%)
Baseline	58.42	47.25	39.18	35.63
+Attribute Reconstruction	79.42	77.53	66.85	65.18
+Degradation Only	83.67	78.92	48.26	46.57
+Full RPT-Mamba	**91.62**	**91.65**	**78.56**	**77.66**

## Data Availability

The experiments are based on the public mTransSee dataset.
